# Comparison of Bacterial Diversity in Air and Water of a Major Urban Center

**DOI:** 10.3389/fmicb.2018.02868

**Published:** 2018-11-29

**Authors:** M. Elias Dueker, Shaya French, Gregory D. O’Mullan

**Affiliations:** ^1^Biology and Environmental & Urban Studies Programs, Bard College, Annandale-on-Hudson, NY, United States; ^2^Bard Center for the Study of Land, Air, and Water, Annandale-on-Hudson, NY, United States; ^3^Cary Institute of Ecosystem Studies, Millbrook, NY, United States; ^4^School of Earth and Environmental Sciences, Queens College, City University of New York, New York City, NY, United States

**Keywords:** aerosol, urban, sewage, microbial exchange, waterfront, diversity

## Abstract

The interaction of wind with aquatic and terrestrial surfaces is known to control the creation of microbial aerosols allowing for their entrainment into air masses that can be transported regionally and globally. Near surface interactions between urban waterways and urban air are understudied but some level of interaction among these bacterial communities would be expected and may be relevant to understanding both urban air and water quality. To address this gap related to patterns of local air-water microbial exchange, we utilized next-generation sequencing of 16S rRNA genes from paired air and water samples collected from 3 urban waterfront sites and evaluated their relative bacterial diversity. Aerosol samples at all sites were significantly more diverse than water samples. Only 17–22% of each site’s bacterial aerosol OTUs were present at every site. These shared aerosol OTUs included taxa associated with terrestrial systems (e.g., *Bacillus*), aquatic systems (e.g., *Planktomarina*) and sewage (e.g., *Enterococcus*). In fact, sewage-associated genera were detected in both aerosol and water samples, (e.g., *Bifidobacterium, Blautia*, and *Faecalibacterium*), demonstrating the widespread influence of similar pollution sources across these urban environments. However, the majority (50–61%) of the aerosol OTUs at each site were unique to that site, suggesting that local sources are an important influence on bioaerosols. According to indicator species analysis, each site’s aerosols harbored the highest percentage of bacterial OTUs statistically determined to uniquely represent that site’s aquatic bacterial community, further demonstrating a local connection between water quality and air quality in the urban environment.

## Introduction

Bacterial aerosols can significantly influence ecology, climate, and public health at both local and globally relevant scales (Fröhlich-Nowoisky et al., 2016). Despite these important impacts, the diversity of bacteria inhabiting atmospheric ecosystems remains poorly constrained in terms of biogeography, the relative importance of specific sources, and even in comparison to the bacterialdiversity encountered in other more frequently studied aquatic and terrestrial habitats. A recent analysis of bacterial sequence diversity and sampling effort by environment shows that aerosols have been the focus of far less sampling effort than aquatic, terrestrial, built, and host-associated environments (Schloss et al., 2016). Without understanding bacterial aerosol diversity it is difficult to determine their functional importance to atmospheric chemistry and cloud formation (Morris et al., 2014; Amato et al., 2017) or the risk of infection (Brodie et al., 2007). Atmospheric transport of bacteria also provides an essential redistribution mechanism for viable microbes and genetic potential between distinct regions and disparate habitats (Polis et al., 1997; Säwström et al., 2016; Mayol et al., 2017) making bacterial aerosols a critical factor for understanding the connections driving diversity, and potentially function, in seemingly separate terrestrial and aquatic habitats (e.g., Prospero et al., 2005). Understanding these bacterial diversity and exchange concepts may be particularly important in relation to public health in crowded urban centers, since they are known to support high viable bacterial aerosol concentrations in the context of highly contaminated terrestrial and aquatic systems (Dueker et al., 2012a).

Like terrestrial and aquatic microbial populations (Martiny et al., 2006; Horner-Devine et al., 2007; Womack et al., 2010), microbial aerosols appear to have distinct geographic patterns determined by source delivery, environmental selection, and geographic distance or isolation. The mechanisms crafting this pattern in microbial aerosols are understudied in both urban and non-urban environments. Bowers et al. (2011a) found that spatial variability in airborne bacterial communities was strongly related to land-use type and source shifts, but not local meteorology- suggesting that source delivery may be a more important driver of bacterial diversity in the atmosphere than environmental growth and selection. Land use patterns and human activity were also found to be an important factor in determining aerosolized urban microbial diversity (Balyan et al., 2017). Microbial assemblages in terrestrial and aquatic microbial communities, on the other hand, appear to be strongly influenced by environmental gradients at the local level (Crump et al., 2004; Van der Gucht et al., 2007; Fierer et al., 2008) suggesting that, perhaps more so than in the atmospheric environment, selection based on environmental conditions is a very important driver of bacterial diversity and composition.

At a small geographic scale, Lee et al. (2017) found that wind speed and wind direction were correlated with high airborne bacterial diversity using 2–3 day moving averages, pointing to proximity of sources and active resuspension as a driver of diversity in urban air. Local meteorological dynamics (wind speed, wind direction) have been shown to aid in the creation of microbial aerosols from local scale aquatic (Dueker et al., 2017) and terrestrial surfaces (Hara and Zhang, 2012). Several studies have also found increased diversity in microbial aerosol samples with low humidity conditions (Lee et al., 2017) and increased wind speeds (Jones and Harrison, 2004). In general, the atmosphere is often assumed to be more homogenous, or less spatially patchy, than aquatic and especially terrestrial habitats in terms of environmental conditions including access to growth substrates, temperature, and UV exposure. For example, estuaries are characterized by spatially complex environmental gradients, while environmental gradients in the near surface air masses over these estuaries can be more homogeneous. The atmosphere is also expected to be more dilute in terms of concentrations of growth substrates and therefore to support less active bacterial communities than most terrestrial and aquatic habitats. How these assumptions about source density, environmental heterogeneity and *in situ* activity change between the urban and rural atmosphere are poorly constrained, as are the consequences for diversity patterns.

Much of the foundational knowledge of microbial aerosol ecology comes from non-urban sites, including remote terrestrial and oceanic regions, sparsely populated coastlines, and mountaintop observatories e.g., (Amato et al., 2007; Fahlgren et al., 2010; Mayol et al., 2017; Evans et al., 2019). This work has determined that microbial aerosol communities reflect inputs from both terrestrial and aquatic systems (Xia et al., 2015). For instance, air masses over remote marine regions reflect long-distance terrestrial inputs, but also local surface water inputs (Xia et al., 2015; Mayol et al., 2017). The primary mechanism for aerosolization of these aquatic bacteria is through bubbles bursting at the water surface (Blanchard and Syzdek, 1971, 1982). Terrestrial aerosols are generally thought to originate from leaves and soil (Bowers et al., 2011a; Šantl-Temkiv et al., 2018) and have been described to primarily contain Gram-positive and spore-forming bacteria, such as *Bacillus sp.* (Griffin et al., 2003; Merrill et al., 2006), while marine aerosols combine long-distance terrestrial influences with a high abundance of Gram-negative bacteria originating from seawater (Pósfai et al., 2003; Cho and Hwang, 2011).

In contrast, our knowledge of microbial diversity and ecology is much less developed in urban regions, especially in the near-surface environment where most microbial exchange and exposure would be expected. The urban environment is thought to harbor distinct microbial aerosols when compared to non-urban areas (Barberán et al., 2015) and likely a greater number and diversity of local suspension sources. Confirmed sources for urban microbial aerosols include terrestrial, aquatic, and regional atmospheric habitats (Asan et al., 2004; Brodie et al., 2007; Fang et al., 2007; Lee et al., 2010; Bowers et al., 2011b; Franzetti et al., 2011; Dueker et al., 2012a, 2017; Ravva et al., 2012; Dueker and O’Mullan, 2014; Balyan et al., 2017). However, environmental controls on urban microbial aerosols, while perhaps less important than in selection-dominated aquatic and terrestrial ecosystems, can’t just be ignored and appear to include season (Bowers et al., 2011a, 2013; Woo et al., 2013; Lee et al., 2017), temperature and humidity (Lee et al., 2017), land use patterns and human activity (Balyan et al., 2017), and, on the local scale, wind direction and wind speed (Montero et al., 2016; Dueker et al., 2017). Some studies have also shown urban aerosols to be influenced by contaminated terrestrial and aquatic environments (Carducci et al., 2000; Pillai and Ricke, 2002; Motta et al., 2008; Dueker et al., 2012a, 2017; Dueker and O’Mullan, 2014).

Despite these studies, our knowledge of urban microbial aerosol communities and their connection to urban aerosolization sources is still quite lacking. To specifically address the potentially bi-directional exchange of bacteria between urban water and air, we simultaneously sampled air and water at 3 urban waterfront sites. This allowed us to evaluate the potential for local connections in waterfront water and air quality. We hypothesized that: (1) the bacterial assemblages in water would be more diverse than in air samples and that air samples would be more uniform across locations, while water would display more local heterogeneity; (2) water and air would harbor distinct dominant bacterial assemblages resulting in greater similarity of water to water, and air to air, across locations; (3) a small subset of taxa would be shared between water and air and when compared among locations, similarity of air to water would be greatest within locations; and (4) urban air would differ from non-urban air, and contain taxa reflecting local sources of urban and, specifically, sewage pollution. While prior cultivation-independent studies have been conducted of both urban water and air separately, to our knowledge this is the first study to simultaneously collect near-surface air and water samples with the purpose of comparing the resulting bacterial community diversity and taxonomic composition.

## Materials and Methods

**FIGURE 1:**
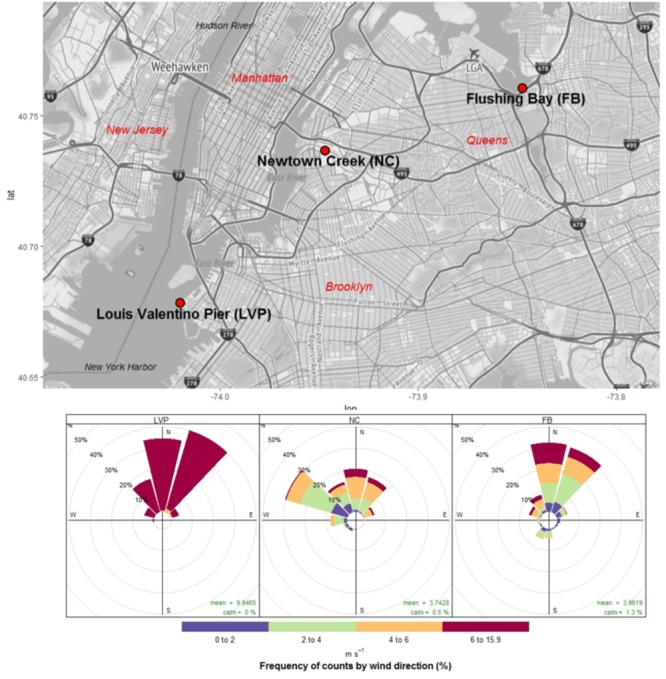
Urban sampling sites (New York, NY, United States) and wind patterns (direction and speed) during sampling events.

Aerosol and surface water samples were collected from three brackish waterfront sites in New York, NY, United States (Figure 1) from Fall 2013 through Summer 2014 (Supplementary Table S1). Flushing Bay (FB) is located in northern Queens, NY, United States (40.761858 N, 73.845919 W), surrounded by industrial and commercial sites and frequently contaminated by raw human sewage delivered through a series of combined sewer outflow (CSO) pipes along its banks (Young et al., 2013). Newtown Creek (NC) is a 3.5-mile East River tributary (40.7368528 N, 73.9464472 W) located on the boundary of Queens and Brooklyn, NY, United States that has been used as a shipping canal since the mid-1800’s. NC was declared an EPA Superfund site in September 2010 and, like FB, receives frequent CSO inputs (Dueker et al., 2012a). Louis Valentino Pier (LVP) is located in Brooklyn, NY, United States on the Southeast shore of NY Harbor (40.67838 N, 74.01966 W) (Dueker et al., 2017). The accessibility of sample sites and presence of onshore winds by site was a constraint on sampling, and resulted in variable sampling by site (Supplementary Table S1). All water and aerosol samples were taken simultaneously, with the exception of 5 aerosol samples at NC which did not have paired water samples (Fall 2013).

Surface water samples were collected as per Dueker et al. (2011), from the top 0.5 m of the water surface, and placed in 2 L acid-washed and autoclaved sample containers. Aerosol samples were collected using a Coriolis-micro sampler (centrifugal impinger, Bertin Inc., Saint-Aubin, France) by collecting air for 60 min at 250 l min^-1^, as per Dueker et al. (2017). A Kestrel 4500BT unit with wind vane adjacent (but upwind) to the aerosol sampler logged 1-min temperature, relative humidity, wind speed, and wind direction measurements during sampling. To control for bacterial contamination, the 1.8 m platform housing the Coriolis unit was sterilized with ethanol before deployment. As per Dueker et al. (2017), initial sampling liquid was sterile, endotoxin and DNA-free HyClone water (GE Healthcare, Troy, NY, United States) with 0.005% (final concentration) Triton-X surfactant added to increase sampling efficiency. Sampling liquid was rehydrated every 10 min with sterile HyClone water to replace evaporation losses. After collection, both surface water and aerosol samples were stored in the dark and on ice until processing in the lab.

Both air and water samples were aseptically filtered through a 0.22 μm Sterivex (Millipore/Sigma, Burlington, MA, United States) filter (500 ml surface water, and 12 ml of impinger liquid). Filters were then stored at -80°C. DNA was extracted from filters using Qiagen/MoBio PowerWater extraction kits (Qiagen, Valencia, CA, United States). To control for kit contamination, we also extracted DNA from two blank filters. Amplicon pyrosequencing was then performed on extracted environmental DNA at Molecular Research DNA labs (^1^MRDNA, Shallowater, TX, United States), using a protocol described by Dowd et al. (2008) to determine bacterial community composition. Briefly, parallel sequencing reactions were prepared from each DNA extraction using the eubacterial primer 27F. The DNA was amplified through a single-step 30-cycle PCR using HotStarTaq Plus Master Mix Kit (Qiagen, Valencia, CA, United States). PCR was performed under the following conditions: 94°C for 3 min, followed by 28 cycles of 94°C for 30 s; 53°C for 40 s and 72°C for 1 min; after which a final elongation step at 72°C for 5 min was performed. All amplicon products were then mixed in equal concentrations, purified using Agencourt Ampure beads (Agencourt Bioscience Corporation, MA, United States), and sequenced using Roche 454 FLX titanium instruments and reagents, following manufacturer’s guidelines.

The resulting sequence files were processed with a custom pipeline based on the Mothur 454 SOP (Schloss et al., 2011). In brief, raw sequence files were denoised using PyroNoise (Quince et al., 2009), then trimmed, requiring a sequence minimum length of 200 and allowing for 1 mismatch to the barcode and 2 mismatches to the primer. Then sequences were aligned against the SILVA reference database. Chimeras were detected and removed using UCHIME (Edgar et al., 2011). OTUs were assigned at the 97% identity threshold, using the average neighbor algorithm, then taxonomically classified using the Mothur-formatted version of the Ribosomal Database Project training set (Cole et al., 2009). Our study was focused on bacteria and all sequences classified as “Chloroplast,” Mitochondria,” “Archaea,” or “unknown” were separated from the bacterial dataset for downstream analyses. To control for contamination, any bacterial OTUs found in blank extractions were removed from downstream analyses. Final sequence files can be found under BioSample accession #’s SAMN10288370 – SAMN10288425 ^2^. For comparison purposes, bacterial sequences detected in water and air at a non-urban coastal site (Maine, United States) using methods described in Dueker et al. (2011) and data previously reported in Evans et al. (2019) were also processed using the above custom pipeline, and were included in some downstream analyses.

Upon completion of the sequence processing, sequences were combined with environmental metadata for analysis in phyloseq (McMurdie and Holmes, 2013), a microbiome analysis package in R Core Team (2018). We ran these analyses on both rarefied and non-rarefied data to test for effects from varying sequencing depths, since aerosol libraries were generally smaller than water surface libraries. We did not remove rare OTUs from these analyses. Alpha and beta-diversity statistics visualizations were acquired using phyloseq and ggplot2 (Wickham, 2009). Venn diagrams were created using the VennDiagram R package (Chen, 2018). Statistical tests of differences in diversity (using Shannon’s H index) and similarity (using Bray-Curtis Dissimilarity) between habitats and sites were performed in the stats (R Core Team (2018)), ggpubr (Kassambara, 2018), and vegan (Oksanen et al., 2017) R packages. Specifically, ANOVA and Tukey *post hoc* analyses were run on multiple-site comparisons and Wilcoxon tests were run on water vs. air comparisons, with statistical signficance assigned at *p* < 0.05. Ordination plots were designed using principal coordinates analysis of a Bray-Curtis Dissimilarity matrix, and euclidean distance calculations were used to construct cluster ellipses. Phylogenetic clustering was evaluated using the Mean Pairwise Distance (selection-based clustering) and Mean Nearest Taxon Distance (abiotic-based selection) indices (PhyloMeasures R Package) (Tsirogiannis and Sandel, 2015; Zhou and Ning, 2017).

DESeq2 was used to identify over-represented taxonomic groups in water vs. air (Love et al., 2014). Indicator species analysis was performed on water samples by site with the indicspecies R package (De Caceres and Legendre, 2009). Aerosol libraries were then interrogated for the presence of water-indicating species as a means to evaluate water surfaces as a source for urban aerosols. Source analysis for sewage-associated bacteria was performed in phyloseq using bacterial types identified in previous literature as representative of both sewage and sewage infrastructure (Brodie et al., 2007; Shao et al., 2009; Wéry et al., 2010; VandeWalle et al., 2012; Cai et al., 2014; Gerritsen et al., 2014; McLellan and Eren, 2014; Barberán et al., 2015; Newton et al., 2015; Supplementary Table S2). After identifying sewage-associated OTUs, representative sequences were chosen using Mothur and BLASTed against the NCBI nucleotide database. Environmental sources for the top 10 hits for each representative sequence of the 50 most abundant OTUs were then recorded to further evaluate sewage-association of these OTUs (Supplementary Table S3).

## Results

Most urban waterfront sampling occurred under onshore wind conditions, which were primarily from the North at all sites (Figure 1). Wind speeds varied from calm to 11 m s^-1^, with highest winds speeds measured at LVP and lowest at NC (Figure 1). Mean air temperature during sampling at these sites ranged from 9.6 to 18.1°C (Supplementary Table S1), while water temperatures ranged from 8.9 to 15.9°C. All surface waters were brackish, with salt concentrations from 17.7 to 20.9 ppt.

**FIGURE 2:**
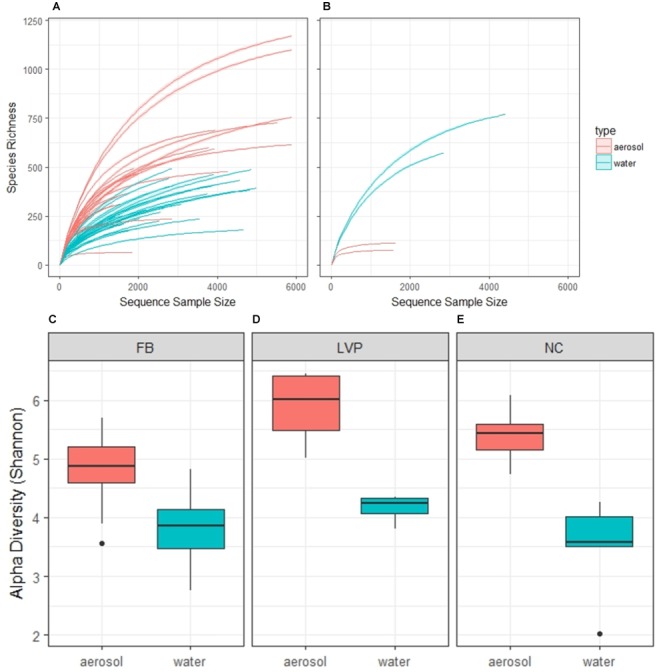
Rarefaction curves by habitat in the urban environment (New York, NY, United States) **(A)** and non-urban environment (Coastal Maine) **(B)**; and alpha diversity estimates of paired aerosol and water samples by site **(C–E)**. Aerosol bacterial communities are significantly more diverse than water at all sites, according to Wilcoxon test (FB: *p* < 0.001, LVP: *p* < 0.05, and NC: *p* < 0.01).

Our sequence quality pipeline yielded a total of 77,262 sequences from 454 pyrosequencing of 16S rRNA in water and aerosol samples. OTU analysis indicated diverse microbial assemblages in both water and air, resulting in 8,927 OTUs (3,648 in water and 6,137 in air) at the level of 97% identity. The aerosol bacterial communities harbored more “rare” OTUs than water bacterial communities, as demonstrated by rank-abundance curves (Supplementary Figure S2). Rarefaction curves (Figure 2A) and alpha diversity estimates (Figures 2C–E) for the urban environment demonstrated that OTU richness and diversity were significantly higher, by site, for aerosols (mean Shannon’s H index: 5.1 ± 0.1) when compared to water samples (mean Shannon’s H index: 3.8 ± 0.1), especially for the high wind aerosol samples taken from LVP (Figure 2D). LVP aerosols were significantly more diverse than FB aerosols, but not NC aerosols (ANOVA, Tukey *post hoc, p* < 0.05). In contrast, water community diversity showed no difference across sites (ANOVA, *p* > 0.05). Analysis performed on rarefied sequence libraries resulted in similar results in terms of relative differences between water and air, but Shannon’s H indices were lower (aerosols: 4.2 ± 0.03, water: 3.1 ± 0.2) (Supplementary Figure S4).

While the pattern of higher diversity in aerosols than water was consistent across the three urban sites, it was quite different from the pattern observed at a non-urban coastal site in Maine, where water was found to have much higher species richness with sampling effort (Figure 2B). By comparison, the aerosol samples from coastal Maine were also less diverse than urban air in this study, while the water samples from coastal Maine were more diverse than urban water from this study (Figure 2B).

**FIGURE 3:**
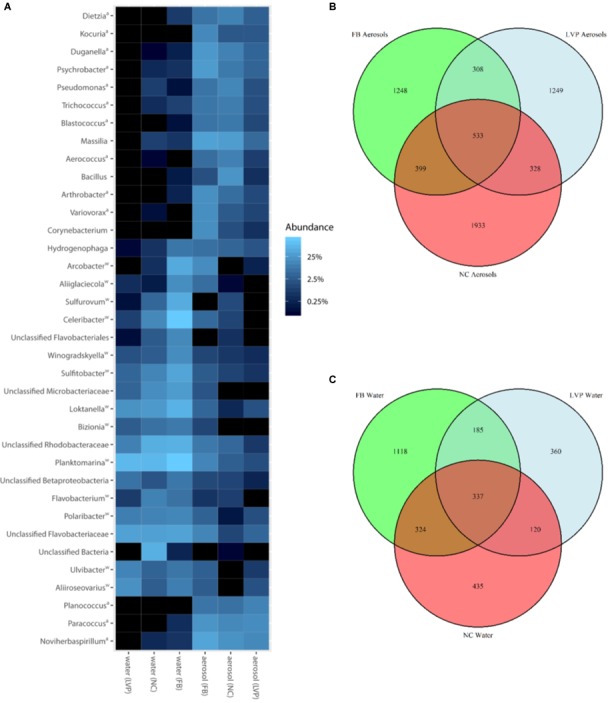
Bacterial communities in surface water and aerosols. **(A)** Dominant top 50 aerosol and water surface OTUs by genus and site. Superscript key: a = taxa belongs to DESeq2-determined over-represented group in aerosols; w = taxa belongs to DESeq2-determined over-represented group in water. **(B)** Aerosol OTU distribution across sites (Flushing Bay (FB) *n* = 25,168 sequences (15 samples); Louis Valentino Pier (LVP) *n* = 20,220 sequences (4 samples); Newtown Creek (NC) *n* = 27,496 sequences (11 samples), **(C)** Water OTU distribution across sites (FB *n* = 37,981 sequences (15 samples); LVP *n* = 14,699 sequences (4 samples); NC *n* = 23,154 sequences (8 samples).

Bacterial community membership for both water and aerosols was highly diverse, dominated at the phylum level by Verrucomicrobia, Proteobacteria, Firmicutes, Bacteroidetes, Actinobacteria, and Acidobacteria (Supplementary Figure S1). Dominant OTUs included bacteria commonly associated with aquatic (e.g., *Planktomarina sp.*), terrestrial (e.g., *Bacilli sp.*), and sewage (*Arcobacter sp., Trichococcus sp.*) sources (Figure 3A and Supplementary Table S2). A total of 46 OTUs were ubiquitous, meaning they were found at all sites and in both water and air habitats (Table 1). These ubiquitous OTUs represented only 5% of the aerosol sequence library but represented 34% of the water sequence library. They included organisms that have commonly been associated with sewage such as *Arcobacter, Romboutsia*, and *Zoogloea* (Table 1 and Supplementary Table S2).

**Table 1:** Taxonomic assignment of OTUs that were detected at all urban sites, and in both air and water habitats at those sites.

**Proteobacteria**		**Actinobacteria**	
Otu00031	Loktanella	Otu00176	Mycobacterium
Otu00027	Loktanella		
Otu00003	Planktomarina	**Bacteroidetes**	
Otu00036	Sulfitobacter	Otu00094	Bacteroides
Otu00097	Unclassified Rhodobacteraceae	Otu00121	Prevotella
Otu00075	Candidatus_ Pelagibacter (SAR11)	Otu00654	Flavobacterium
Otu00606	Sphingorhabdus	Otu00180	Flavobacterium
Otu00076	Unclassified Alphaproteobacteria	Otu00619	Flavobacterium
Otu00590	Unclassified Alcaligenaceae	Otu00251	Flavobacterium
Otu00965	Caenimonas	Otu00156	Flavobacterium
Otu00153	Hydrogenophaga	Otu00527	Flavobacterium
Otu00138	Hydrogenophaga	Otu00070	Polaribacter
Otu00297	Polaromonas	Otu00185	Ulvibacter
Otu00287	Rhodoferax	Otu00023	Unclassified Flavobacteriaceae
Otu00116	Simplicispira	Otu00122	Unclassified Flavobacteriaceae
Otu00526	Unclassified Comamonadaceae	Otu00039	Unclassified Flavobacteriaceae
Otu00200	Rivicola	Otu00044	Winogradskyella
Otu00361	Zoogloea	Otu00762	Unclassified Flavobacteriales
Otu00109	Unclassified Betaproteobacteria	
Otu00055	Arcobacter	**Firmicutes**	
Otu00680	Arcobacter	Otu00234	Romboutsia
Otu00510	Arcobacter		
Otu00189	Arcobacter	**Fusobacteria**	
Otu00017	Arcobacter	Otu00390	Unclassified Fusobacteriaceae
Otu00040	Aeromonas		
Otu00029	Tolumonas		
Otu00096	Tolumonas		
Otu00282	Rhizobacter		

Dominant aerosol OTUs were typically shared across sites and found in similar relative abundances (Figure 3A), however, the majority of aerosol OTUs were unique to site (Figure 3B). In water, dominant OTUs were often shared and had similar relative abundances, but a smaller portion of OTUs were unique to sites in water, as compared to aerosols (Figures 3B,C). In aerosols, 533 OTUs (representing 9% of the aerosol sequence library) were found at all three sites. LVP aerosols harbored the highest percentage of shared OTUs (22%) and NC aerosols had the least (17%). For water surfaces, 337 OTUs (representing 12% of the water sequence library) were shared among all three sites. LVP water harbored the highest percentage of shared water OTUs (34%) and FB water had the least shared OTUs (17%).

**FIGURE 4:**
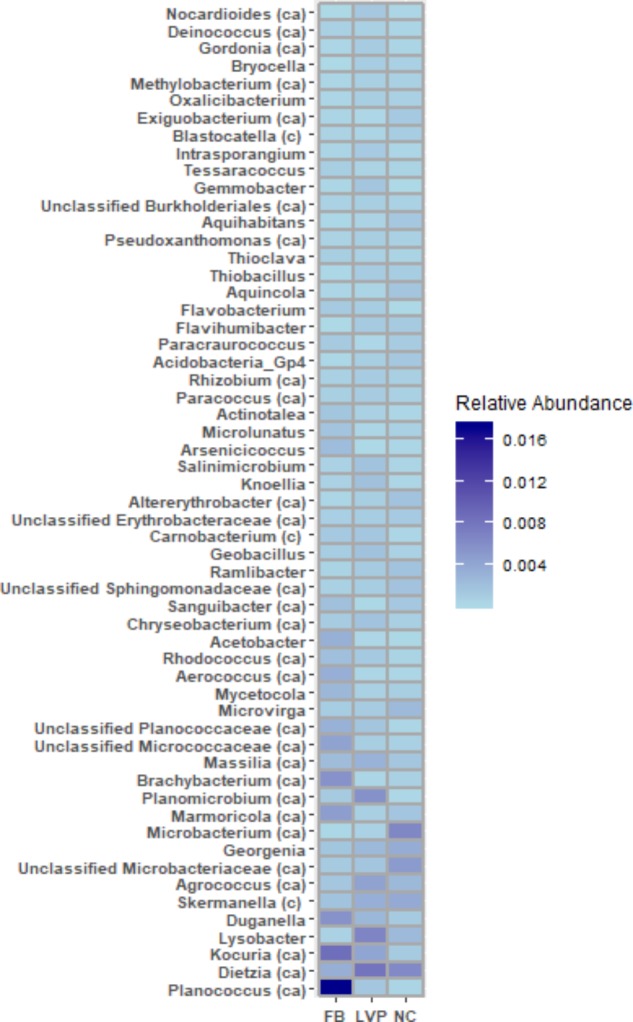
OTUs (collapsed by genera and limited to those representing >0.2% of total library) unique to air (not found in water) but shared across all 3 sites. c = present in cloud DNA, ca = present in cloud DNA and RNA (Amato et al., 2017).

**FIGURE 5:**
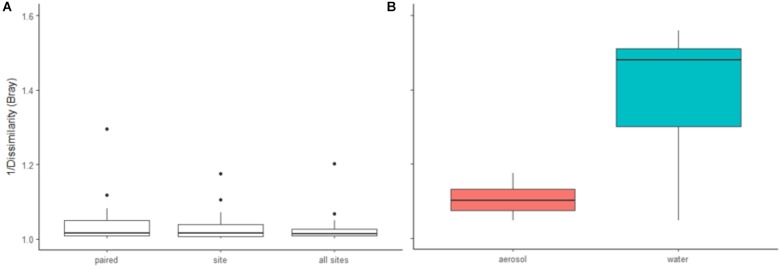
Similarity analyses (1/Bray dissimilarity) of **(A)** individual aerosol samples compared to water samples (paired samples, unpaired samples (by site), all sites pooled), and **(B)** aerosols to aerosols and water to water at each site. [**A**: no significant difference (ANOVA), **B**: *p* < 0.0001 (Wilcoxon test)].

**FIGURE 6:**
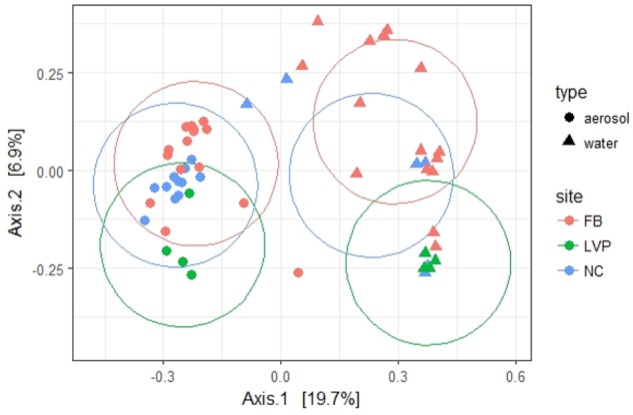
Beta diversity [principal coordinates (multidimensional scaling) analysis] of water and aerosol samples across sites. Percentages in axes represent % of variation explained by that axis. Ellipses calculated using Euclidean distance (ggplot2, R package).

Of the 533 aerosol OTUs shared across sites (Figure 2B), 164 (31%) were found only in aerosols, and not in water. These aerosol-unique OTUs represented 10% of the entire aerosol library, with relatively even representation across sites (11% of FB, 10% of LVP, and 9% of NC). However, the relative abundance of individual aerosol-unique OTUs did vary across sites (Figure 4). Overall, dominant OTUs from water occurred at very different frequencies in aerosol samples, and vice versa (Figure 3A), causing the bacterial assemblage in air to be more similar across sites, than water to air at a single site (Figures 5A,B). Water samples had significantly higher phylogenetic diversity within samples than aerosols, providing evidence for stronger selection on microbial communities in water as opposed to air (Supplementary Figures S5, S6) (*p* < 0.01). However, it’s worth noting that aerosols also had clustering results indicating some level of selection within site (Supplementary Figures S5, S6). Water samples were significantly more similar to other water samples than aerosols were to other aerosol samples (Figure 5B, Wilcoxon test, *p* < 0.05). When considering the entire bacterial assemblage. a similar pattern was found, with distinct clusters evident by site and habitat type (Figure 6) using principal coordinates analysis and the Bray-Curtis Dissimilarity Index.

**FIGURE 7:**
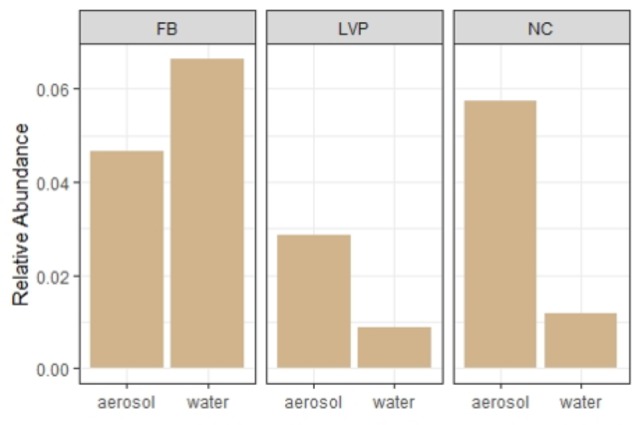
Relative abundance of sewage-associated bacterial sequences in water and air at each site.

Water and air samples shared key taxa and showed signs of sewage contamination. Differential abundance analysis (DESeq2) identified distinct over-represented taxa in water and aerosol libraries (Supplementary Figure S3), which were present in the dominant taxa (Figure 2A), the aerosol-unique library (Figure 4) and the ubiquitous (all sites, all habitats) library (Table 1). Similarly, indicator species analysis identified OTUs deemed to be statistically characteristic of each site. These water-indicating OTUs were found to be more prevalent in the matching site’s aerosols as compared to other site’s aerosols (Table 2). Sewage-associated bacteria were found in both water and air at all sites (Figure 7). Representative sequences for dominant sewage-associated OTUs confirmed sewage association assumptions through BLAST results (Supplementary Table S3), having also been detected in sewage sludge, human feces, wastewater, feces contaminated river water, and other sewage-related sources. Aerosol libraries contained a higher relative abundance of sewage-associated bacteria (∼1%) than water surfaces (∼0.3%). All sewage-associated bacteria found in water samples were also detected in aerosols, including *Bacteroides, Faecalibacterium, Trichococcus, Blautia, Bifidobacterium*, and *Aeromonas* (Supplementary Table S2). Aerosols additionally contained *Anaerofilum, Clostridium, Butyrivibrio, Dorea, Dysgonomonas*, and *Ruminococcus* (Supplementary Table S2).

**Table 2:** Percentages of water-specific indicator OTU’s in aerosol libraries.

	FB Air	LVP Air	NC Air
FB Water	0.0126	0.0003	0.0000
LVP Water	0.0121	0.0039	0.0005
NC Water	0.0011	0.0002	0.0011

*Note that air at each site harbors more of its own water’s indicators than it does of other site’s indicators*.

## Discussion

### Urban Bacterial Aerosols Are More Diverse Than Urban Surface Water Bacterial Communities

In general, diversity of microbial aerosols is thought to reflect dominant source ecology more than environmental selection (Bowers et al., 2013). Since air is a combination of all surface sources (aquatic and terrestrial) and its own community, we would expect diversity of bacterial aerosols to reflect the diversity of these systems combined. The soil environment is known to be heterogeneous, rich in substrates, and supports the highest bacterial diversity relative to sampling effort when compared to other habitats (Quince et al., 2008). Aquatic environments, which are sometimes viewed as more environmentally homogeneous than soil, harbor bacterial communities that have been found to be less diverse than soil relative to sampling effort (Schloss et al., 2016), however, estuarine environments, relative to some other aquatic systems, are known to have complex environmental gradients and sharp transitions in diversity (Crump et al., 2004). It would follow, then, that air, a much more dilute fluid (and presumably much better mixed) than water, might harbor less diverse and more homogenized bacterial communities than found in water. This remains largely speculative since the atmosphere has been the focus of far less sampling effort (Schloss et al., 2016). In addition, soil and coastal waterways are generally considered to harbor highly active microbial communities subject to potentially high rates of growth and selection (e.g., Crump et al., 2004), while atmospheric environments harbor less active communities and might be expected to have less spatially variable selection dynamics. Constrained activity and selection, combined with the assumption of more spatially homogeneous environmental conditions in the lower atmosphere, lead to our expectation of lower diversity and more spatially homogenous patterns of diversity in the urban atmosphere compared to urban waterways.

Bacterial aerosols in this study were consistently more diverse than water surface communities from the same local urban environment, contrary to our initial expectation. This was in spite of the fact that most aerosol libraries were smaller than water libraries. Furthermore, urban aerosols harbored rarer OTUs than water surfaces, suggesting that the rare biosphere plays a major role in aerosol diversity. Perhaps the high levels of diversity should not be surprising, as Womack et al., 2010 argued that despite common perceptions air must be considered a diverse and active microbial habitat. They also pointed out the lack of data to inform these assumptions and the infancy of microbial diversity and biogeography studies relative to terrestrial and aquatic habitats.

Our study provides a unique pairwise comparison of urban air and water and supports the important role of air as a microbial habitat in need of additional study. Despite the higher diversity of microbial communities in air, water samples in our study were found to be less similar across locations (Figure 5B) than air, with water displaying greater clustering within samples than air (Supplementary Figures S4, S5). This is consistent with stronger selection, responding to greater spatial heterogeneity in environmental conditions, in water as compared to air. The relative influence of selection in water vs. air is of great interest and not yet clearly resolved in the literature. Additionally, the phylogenetic clustering in air, while less than in water, does still suggest a role of selection in air and is again consistent with Womack et al., 2010 view of air as a living habitat.

The primary goal of this study was to provide a relative comparison of diversity in paired water and air samples from New York City, however, as there have been relatively few bacterial aerosol studies focused on diversity patterns it is also useful to compare these results to prior studies. Caution must be used, however, in comparing diversity across studies, as the relative sampling effort and methodological approaches can create important constraints on these comparisons. The mean Shannon’s H for urban waterways in our study was lower than previously reported diversity analyses of urban river waters in the Zenne River (Brussels, Belgium) (García-Armisen et al., 2014), but similar to those reported from polluted sites along the Santa Ana River Watershed (California, United States) (Ibekwe et al., 2016). The bacterial diversity of urban aerosols in this study was more than 2 times higher than those previously reported in marine aerosols (Xia et al., 2015), non-urban coastal waterfront aerosols (Fahlgren et al., 2010), and in the pm 2.5 fraction of urban, rural, and high-alpine air (Despres et al., 2007). These data, including the high percentage of aerosol OTUs that were unique by site, suggests that there are diverse and important aerosol sources from the local urban environment (near-surface, short-distance transport) and reinforces the view of air as an important microbial habitat and reservoir of substantial genetic diversity.

In addition to sampling effort and amplicon sequencing methods, the differences in reported bacterial aerosol diversity from our study compared to prior literature may be partially related to sampling height. The high diversity of urban near-surface bacterial aerosols likely relies heavily on local mechanical interactions that create large particles with short residence times, and therefore bacteria associated with these larger particles wouldn’t be detected by sampling conducted at higher altitudes. Our sampling was conducted at 1.8 m, whereas other reported aerosol samples were conducted from a tower (Fahlgren et al., 2010), high altitudes (Despres et al., 2007), and from atop a research vessel (Xia et al., 2015). As it is assumed that surface suspension is a driving factor in bacterial aerosol concentrations, it is not surprising that near surface sampling may yield high diversity. This reinforces the importance of near-surface processes in understanding aerosol dynamics. In the waterfront environments included in this study, the terrestrial-air-water interface may itself create an “edge effect,” setting up an ecotone in near surface air that may harbor a higher diversity of bacteria than the water itself and the higher-altitude air above it. Interestingly, studies of bacterial community structure and membership in the atmosphere display a similar pattern (just in reverse) to depth patterns identified in the oceans., In oceanic systems, near-surface bacterial communities appear to be structured through different selection processes than those in deep ocean water (Ghiglione et al., 2012). The same may be true in the atmosphere, with boundary-layer microbial communities facing different selection pressures than free troposphere communities are experiencing (Zweifel et al., 2012).

Since differences in diversity may be related to local sources, as well as selection driven by environmental heterogeneity, it is perhaps not surprising that higher diversity would be observed in urban aerosols where a large number of distinct aerosol sources would be expected. For example, urban aerosol diversity was higher than samples retrieved from a coastal Maine waterfront and in other waterfront sampling (e.g., Fahlgren et al., 2010). Sources require suspension mechanisms, so it would be expected that levels of bacterial diversity in urban aerosols may significantly change with environmental conditions that control aerosolization from surfaces, including wind speed (Montero et al., 2016; Dueker et al., 2017). In this study, wind speed does seem to have contributed to the higher bacterial aerosol diversity observed at the LVP site and may contribute to the observed heterogeneity across sites.

### Urban Water and Aerosol Bacterial Communities Are Distinct

Bacterial communities in water and air in this megacity were dominated by 6 phyla: Verrucomicrobia, Proteobacteria, Firmicutes, Bacteroidetes, Actinobacteria, and Acidobacteria. These phyla are commonly reported for urban microbial aerosols (e.g., Lee et al., 2017). The ubiquity of common soil and aquatic bacteria in water and air across all sites suggests a strong bacterial exchange component between water, air, and terrestrial systems in the urban environment. For instance, *Planktomarina, Aliiroseovarius*, and SAR11, common water bacteria, were present in both the water and the air. *Bacillus* and *Microbacteraceae*, common soil organisms, were also found in both. Despite these common sources, urban air and water bacterial communities were still quite distinct, both from each other and from non-urban waterfront aerosols. In the urban environment, water samples had bacterial communities that were more similar to each other than air samples. This, again, is surprising given the assumption that water would be less homogeneous than air in terms of mixing. The taxonomic diversity of air was certainly not low in diversity or highly homogeneous, as initially expected. The majority of aerosol OTUs were unique to a site. This suggests that substantial effort will be required to understand the diversity of urban air, and that the connections between bacterial diversity and function from a geochemical or public health perspective will be difficult to constrain due to the potential of the rare OTUs in urban air to have functional significance.

Atmospheric environments appear to have a high capacity for environmental selection, rather than just local source delivery, to influence the observed aerosol diversity. We found that many genera unique to urban aerosols (and present at all sites) were also recently found to be present and metabolically active in clouds (Amato et al., 2017; Table 1 and Figure 4A), suggesting that these bacterial genera may constitute a distinct bacterial aerosol community. Interestingly, our near-surface aerosol samples were lower in bacterial diversity than those detected in clouds (Amato et al., 2017), at high altitude, which may have to do with the singular habitat that clouds provide to bacterial aerosols. In the near-surface environment, fog plays a similar role, promoting viability and increased diversity of bacterial aerosols (Dueker et al., 2012b). Our non-foggy urban aerosols were clearly more diverse than rural non-foggy Coastal Maine aerosols, but when fog was present in coastal Maine, bacterial aerosol community diversity matched our urban aerosol sample diversity (Evans et al., 2019).

### Local Contamination of Water and Air Affects Air Quality in the Urban Environment

Near-surface air and water surfaces are known to share bacterial communities (Dueker et al., 2017). Urban waterways may function as sources for bacterial aerosol concentrations and diversity. Many of the ubiquitous bacteria in this study (present at all urban sites, in both water and air) were clearly of aquatic origin, as were many of the dominant OTUs present in water and air in the urban environment. This could be due to long-distance transport and influences from the ocean and remote aquatic surfaces (Mayol et al., 2017), but results from the DESeq2 and Indicator Species analyses (outlining dominance and presence of habitat-distinct taxa in both water and air) indicate strong influences on bacterial aerosols from local aquatic sources as well.

In crowded urban centers, the aquatic environment is frequently contaminated with sewage (treated and untreated), and therefore may be a source of sewage contamination (including pathogenic bacteria and viruses) to urban air. Once aerosolized, sewage microbes can move from water to air, and from air to water, although the original source of sewage contamination is wastewater (O’Mullan et al., 2017). In this study, we found evidence of sewage contamination in both water and air, at all sites, demonstrating a local connection between water quality and air quality in the urban environment. Furthermore, urban aerosols contained sewage-associated bacteria that were not reflected in the local waterways –suggesting sewage sources other than local contaminated waterways. These aerosolization sources could include nearby waste treatment plants (Brandi et al., 2000; Heinonen-Tanski et al., 2009; Gangamma et al., 2011), aerosolized biosolids (Baertsch et al., 2007), or animal waste (Bowers et al., 2011b).

To our knowledge, this is the first study to compare simultaneously collected water and air samples in an urban environment on the local scale using amplicon sequencing tools. Our results support the view of Womack et al. (2010) of air as a rich microbial habitat, not just a conduit for transport, with the potential to harbor diversity greater than some terrestrial and aquatic habitats. Given our findings, including higher bacterial diversity in urban aerosols than in urban water, it is clear that more research on this scale should be conducted to better understand the extent to which bacterial exchange occurs between urban water and air, and under what conditions. This research will greatly expand our current grasp not only on urban bacterial biogeography but also our ability to understand human health implications from this distribution, and to make management decisions that mitigate risk.

## Author Contributions

MD and GO’M designed and conducted the study. SF performed bioinformatics and organized the environmental database. MD and SF conducted statistical analyses and wrote the first draft of the manuscript. GO’M wrote sections of the manuscript.

## Conflict of Interest Statement

The authors declare that the research was conducted in the absence of any commercial or financial relationships that could be construed as a potential conflict of interest. The reviewer IM and handling Editor declared their shared affiliation.

## Footnotes

1 www.mrdnalab.com

2 www.ncbi.nlm.nih.gov/biosample

## Abbreviations

FB: Flushing Bay
LVP: Louis Valentino Pier
NC: Newtown Creek

## References

[B1] Amato P. Joly M. Besaury L. Oudart A. Taib N. Moné A. I. (2017). Active microorganisms thrive among extremely diverse communities in cloud water. *PLoS One* 12:e0182869. 10.1371/journal.pone.0182869 28792539 PMC5549752

[B2] Amato P. Parazols M. Sancelme M. Mailhot G. Laj P. Delort A. M. (2007). An important oceanic source of micro-organisms for cloud water at the Puy de Dome (France). *Atmos Environ.* 41 8253–8263. 10.1016/j.atmosenv.2007.06.022

[B3] Asan A. Ilhan S. Sen B. Erkara I. P. Filik C. Cabuk A. (2004). Airborne fungi and actinomycetes concentrations in the air of Eskisehir city (Turkey). *Indoor Built Environ.* 13 63–74. 10.1177/1420326X04033843

[B4] Baertsch C. Paez-Rubio T. Viau E. Peccia J. (2007). Source tracking aerosols released from land-applied class B biosolids during high-wind events. *Appl. Environ. Microbiol.* 73 4522–4531. 10.1128/AEM.02387-06 17513591 PMC1932808

[B5] Balyan P. Ghosh C. Das S. Banerjee B. D. (2017). Spatial variation of biogenic aerosols at different land use configurations in urban Delhi. *Int. J. Appl. Environ. Sci.* 12 731–744.

[B6] Barberán A. Ladau J. Leff J. W. Pollard K. S. Menninger H. L. Dunn R. R. (2015). Continental-scale distributions of dust-associated bacteria and fungi. *Proc. Natl. Acad. Sci. U.S.A.* 112:5756. 10.1073/pnas.1420815112 25902536 PMC4426398

[B7] Blanchard D. C. Syzdek L. (1971). Bubbles and water-to air transfer of bacteria. *Bull. Am. Meteorol. Soc.* 52 1136–1141.

[B8] Blanchard D. C. Syzdek L. D. (1982). Water-to-air transfer and enrichment of bacteria in drops from bursting bubbles. *Appl. Environ. Microbiol.* 43 1001–1005. 16346001 10.1128/aem.43.5.1001-1005.1982 PMC244176

[B9] Bowers R. M. Clements N. Emerson J. B. Wiedinmyer C. Hannigan M. P. Fierer N. (2013). Seasonal variability in bacterial and fungal diversity of the near-surface atmosphere. *Environ. Sci. Technol.* 47 12097–12106. 10.1021/es402970s 24083487

[B10] Bowers R. M. McLetchie S. Knight R. Fierer N. (2011a). Spatial variability in airborne bacterial communities across land-use types and their relationship to the bacterial communities of potential source environments. *ISME J.* 5 601–612. 10.1038/ismej.2010.167 21048802 PMC3105744

[B11] Bowers R. M. Sullivan A. P. Costello E. K. Collett J. L. Knight R. Fierer N. (2011b). Sources of bacteria in outdoor air across cities in the Midwestern United States. *Appl. Environ. Microbiol.* 77 6350–6356. 10.1128/AEM.05498-11 21803902 PMC3187178

[B12] Brandi G. Sisti M. Amagliani G. (2000). Evaluation of the environmental impact of microbial aerosols generated by wastewater treatment plants utilizing different aeration systems. *J. Appl. Microbiol.* 88 845–852. 10.1046/j.1365-2672.2000.01024.x 10792545

[B13] Brodie E. L. DeSantis T. Z. Parker J. P. M. Zubietta I. X. Piceno Y. M. Andersen G. L. (2007). Urban aerosols harbor diverse and dynamic bacterial populations. *Proc. Natl. Acad. Sci. U.S.A.* 104 299–304. 10.1073/pnas.0608255104 17182744 PMC1713168

[B14] Cai L. Ju F. Zhang T. (2014). Tracking human sewage microbiome in a municipal wastewater treatment plant. *Appl. Microbiol. Biotechnol.* 98 3317–3326. 10.1007/s00253-013-5402-z 24305737

[B15] Carducci A. Tozzi E. Rubulotta E. Casini B. Cantiani L. Rovini E. (2000). Assessing airborne biological hazard from urban wastewater treatment. *Water Res.* 34 1173–1178. 10.1016/S0043-1354(99)00264-X

[B16] Chen H. (2018). *VennDiagram: Generate High-Resolution Venn and Euler Plots. R Package version 1620*. Available at: https://rdrr.io/cran/VennDiagram/

[B17] Cho B. C. Hwang C. Y. (2011). Prokaryotic abundance and 16S rRNA gene sequences detected in marine aerosols on the East Sea (Korea). *FEMS Microbiol. Ecol.* 76 327–341. 10.1111/j.1574-6941.2011.01053.x 21255051

[B18] Cole J. R. Wang Q. Cardenas E. Fish J. Chai B. Farris R. J. (2009). The Ribosomal Database Project: improved alignments and new tools for rRNA analysis. *Nucleic Acids Res.* 37 D141–D145. 10.1093/nar/gkn879 19004872 PMC2686447

[B19] Crump B. C. Hopkinson C. S. Sogin M. L. Hobbie J. E. (2004). Microbial biogeography along an estuarine salinity gradient: combined influences of bacterial growth and residence time. *Appl. Environ. Microbiol.* 70 1494–1505. 10.1128/AEM.70.3.1494-1505.2004 15006771 PMC365029

[B20] De Caceres M. Legendre P. (2009). Associations between species and groups of sites: indices and statistical inference. *Ecology* 90 3566–3574. 10.1890/08-1823.1 20120823

[B21] Despres V. R. Nowoisky J. F. Klose M. Conrad R. Andreae M. O. Poschl U. (2007). Characterization of primary biogenic aerosol particles in urban, rural, and high-alpine air by DNA sequence and restriction fragment analysis of ribosomal RNA genes. *Biogeosciences* 4 1127–1141. 10.5194/bg-4-1127-2007

[B22] Dowd S. E. Callaway T. R. Wolcott R. D. Sun Y. McKeehan T. Hagevoort R. G. (2008). Evaluation of the bacterial diversity in the feces of cattle using 16S rDNA bacterial tag-encoded FLX amplicon pyrosequencing (bTEFAP). *BMC Microbiol.* 8:125. 10.1186/1471-2180-8-125 18652685 PMC2515157

[B23] Dueker M. E. O’Mullan G. D. (2014). Aeration remediation of a polluted waterway increases near-surface coarse and culturable microbial aerosols. *Sci. Total Environ.* 15 184–189. 10.1016/j.scitotenv.2014.01.092 24531127

[B24] Dueker M. E. O’Mullan G. D. Juhl A. R. Weathers K. C. Uriarte M. (2012a). Local environmental pollution strongly influences culturable bacterial aerosols at an urban aquatic Superfund site. *Environ. Sci. Technol.* 46 10926–10932. 10.1021/es301870t 22954203

[B25] Dueker M. E. O’Mullan G. D. Weathers K. C. Juhl A. R. Uriarte M. (2012b). Coupling of fog and marine microbial content in the near-shore coastal environment. *Biogeosciences* 9 803–813. 10.5194/bg-9-803-2012

[B26] Dueker M. E. O’Mullan G. D. Martinez J. Juhl A. R. Weathers K. C. (2017). Onshore wind speed modulates microbial aerosols along an urban waterfront. *Atmosphere* 08:215 10.3390/atmos8110215

[B27] Dueker M. E. Weathers K. C. O’Mullan G. D. Juhl A. R. Uriarte M. (2011). Environmental controls on coastal coarse aerosols: implications for microbial content and deposition in the near-shore environment. *Environ. Sci. Technol.* 45 3386–3392. 10.1021/es1035128 21428380

[B28] Edgar R. C. Haas B. J. Clemente J. C. Quince C. Knight R. (2011). UCHIME improves sensitivity and speed of chimera detection. *Bioinformatics* 27 2194–2200. 10.1093/bioinformatics/btr381 21700674 PMC3150044

[B29] Evans S. E. Dueker M. E. Logan J. R. Weathers K. C. (2019). The biology of fog: results from coastal maine and namib desert reveal common drivers of fog microbial composition. *Sci. Total Environ.* 647 1547–1556. 10.1016/j.scitotenv.2018.08.045 30180359

[B30] Fahlgren C. Hagstrom A. Nilsson D. Zweifel U. L. (2010). Annual variations in the diversity, viability, and origin of airborne bacteria. *Appl. Environ. Microbiol.* 76 3015–3025. 10.1128/AEM.02092-09 20228096 PMC2863461

[B31] Fang Z. G. Ouyang Z. Y. Zheng H. Wang X. K. Hu L. F. (2007). Culturable airborne bacteria in outdoor environments in Beijing, China. *Microb. Ecol.* 54 487–496. 10.1007/s00248-007-9216-3 17308950

[B32] Fierer N. Liu Z. Z. Rodriguez-Hernandez M. Knight R. Henn M. Hernandez M. T. (2008). Short-term temporal variability in airborne bacterial and fungal populations. *Appl. Environ. Microbiol.* 74 200–207. 10.1128/AEM.01467-07 17981945 PMC2223228

[B33] Franzetti A. Gandolfi I. Gaspari E. Ambrosini R. Bestetti G. (2011). Seasonal variability of bacteria in fine and coarse urban air particulate matter. *Appl. Microbiol. Biotechnol.* 90 745–753. 10.1007/s00253-010-3048-7 21184061

[B34] Fröhlich-Nowoisky J. Kampf C. J. Weber B. Huffman J. A. Pöhlker C. Andreae M. O. (2016). Bioaerosols in the earth system: climate, health, and ecosystem interactions. *Atmos. Res.* 182 346–376. 10.1016/j.atmosres.2016.07.018

[B35] Gangamma S. Patil R. S. Mukherji S. (2011). Characterization and proinflammatory response of airborne biological particles from wastewater treatment plants. *Environ. Sci. Technol.* 45 3282–3287. 10.1021/es103652z 21425829

[B36] García-Armisen Tİnceoǧlu Ö Ouattara NK Anzil A Verbanck Brion M. A. N. (2014). Seasonal variations and resilience of bacterial communities in a sewage polluted urban river. *PLoS One* 9:e92579. 10.1371/journal.pone.0092579 24667680 PMC3965440

[B37] Gerritsen J. Fuentes S. Grievink W. van Niftrik L. Tindall B. J. Timmerman H. M. (2014). Characterization of *Romboutsia ilealis* gen. nov., sp. nov., isolated from the gastro-intestinal tract of a rat, and proposal for the reclassification of five closely related members of the genus Clostridium into the genera *Romboutsia* gen. nov., *Intestinibacter* gen. nov., *Terrisporobacter* gen. nov. and *Asaccharospora* gen. nov. *Int. J. Syst. Evol. Microbiol.* 64 1600–1616. 10.1099/ijs.0.059543-0 24480908

[B38] Ghiglione J.-F. Galand P. E. Pommier T. Pedrós-Alió C. Maas E. W. Bakker K. (2012). Pole-to-pole biogeography of surface and deep marine bacterial communities. *Proc. Nat. Acad. Sci.* *U.S.A.* 109:17633. 10.1073/pnas.1208160109 23045668 PMC3491513

[B39] Griffin D. W. Kellogg C. A. Garrison V. H. Lisle J. T. Borden T. C. Shinn E. A. (2003). Atmospheric microbiology in the northern Caribbean during African dust events. *Aerobiologia* 19 143–157. 10.1023/B:AERO.0000006530.32845.8d

[B40] Hara K. Zhang D. Z. (2012). Bacterial abundance and viability in long-range transported dust. *Atmos. Environ.* 47 20–25. 10.1016/j.atmosenv.2011.11.050 26733988

[B41] Heinonen-Tanski H. Reponen T. Koivunen J. (2009). Airborne enteric coliphages and bacteria in sewage treatment plants. *Water Res.* 43 2558–2566. 10.1016/j.watres.2009.03.006 19345977

[B42] Horner-Devine M. C. Silver J. M. Leibold M. A. Bohannan B. J. M. Colwell R. K. Fuhrman J. A. (2007). A comparison of taxon co-occurrence patterns for macro- and microorganisms. *Ecology* 88 1345–1353. 10.1890/06-0286 17601127

[B43] Ibekwe A. Ma J. Murinda S. (2016). Bacterial community composition and structure in an urban river impacted by different pollutant sources. *Sci. Total Environ.* 56 1176–1185. 10.1016/j.scitotenv.2016.05.168 27267715

[B44] Jones A. M. Harrison R. M. (2004). The effects of meteorological factors on atmospheric bioaerosol concentrations – A review. *Sci. Total Environ.* 326 151–180. 10.1016/j.scitotenv.2003.11.021 15142773

[B45] Kassambara A. (2018). *ggpubr: ‘ggplot2’ Based Publication Ready Plots. R Package Version 0.1.7*. Available at: http://www.sthda.com/english/rpkgs/ggpubr

[B46] Lee J. Y. Park E. H. Lee S. Ko G. Honda Y. Hashizume M. (2017). Airborne bacterial communities in three east asian cities of China, South Korea, and Japan. *Sci. Rep.* 17:5545. 10.1038/s41598-017-05862-4 28717138 PMC5514139

[B47] Lee S. H. Lee H. J. Kim S. J. Lee H. M. Kang H. Kim Y. P. (2010). Identification of airborne bacterial and fungal community structures in an urban area by T-RFLP analysis and quantitative real-time PCR. *Sci. Total Environ.* 408 1349–1357. 10.1016/j.scitotenv.2009.10.061 19913878

[B48] Love M. I. Huber W. Anders S. (2014). Moderated estimation of fold change and dispersion for RNA-seq data with DESeq2. *Genome Biol.* 05:550. 10.1186/s13059-014-0550-8 25516281 PMC4302049

[B49] Martiny J. B. H. Bohannan B. J. M. Brown J. H. Colwell R. K. Fuhrman J. A. Green J. L. (2006). Microbial biogeography: putting microorganisms on the map. *Nat. Rev. Microbiol.* 4 102–112. 10.1038/nrmicro1341 16415926

[B50] Mayol E. Arrieta J. M. Jiménez M. A. Martínez-Asensio A. Garcias-Bonet N. Dachs J. (2017). Long-range transport of airborne microbes over the global tropical and subtropical ocean. *Nat. Commun.* 8:201. 10.1038/s41467-017-00110-9 28779070 PMC5544686

[B51] McLellan S. L. Eren A. M. (2014). Discovering new indicators of fecal pollution. *Trends Microbiol.* 22 697–706. 10.1016/j.tim.2014.08.002 25199597 PMC4256112

[B52] McMurdie P. J. Holmes S. (2013). phyloseq: an R package for reproducible interactive analysis and graphics of microbiome census data. *PLoS One* 8:e61217. 10.1371/journal.pone.0061217 23630581 PMC3632530

[B53] Merrill L. Dunbar J. Richardson J. Kuske C. R. (2006). Composition of *Bacillus* species in aerosols from 11 U.S. cities. *J. Forensic Sci.* 51 559–565. 10.1111/j.1556-4029.2006.00132.x 16696702

[B54] Montero A. Dueker M. E. O’Mullan G. D. (2016). Culturable bioaerosols along an urban waterfront are primarily associated with coarse particles. *PeerJ* 4:e2827. 10.7717/peerj.2827 28028485 PMC5182991

[B55] Morris C. E. Conen F. Alex Huffman J. Phillips V. Pöschl U. Sands D. C. (2014). Bioprecipitation: a feedback cycle linking Earth history, ecosystem dynamics and land use through biological ice nucleators in the atmosphere. *Glob Change Biol.* 20 341–351. 10.1111/gcb.12447 24399753

[B56] Motta O. Capunzo M. De Caro F. Brunetti L. Santoro E. Farina A. (2008). New approach for evaluating the public health risk of living near a polluted river. *J. Prevent. Med. Hygiene* 49 79–88. 18847182

[B57] Newton R. J. McLellan S. L. Dila D. K. Vineis J. H. Morrison H. G. Eren A. M. (2015). Sewage reflects the microbiomes of human populations. *MBio* 6 e2574-14. 10.1128/mBio.02574-14 25714718 PMC4358014

[B58] Oksanen J. Guillaume Blanchet F. Friendly M. Kindt P. Legendre P. McGlinn D. (2017). *Vegan: Community Ecology Package. R Package Version 24-3*. Available at: https://CRAN.R-project.org/package=vegan

[B59] O’Mullan G. D. Dueker M. E. Juhl A. R. (2017). Challenges to managing microbial fecal pollution in coastal environments: extra-enteric ecology and microbial exchange among water, sediment, and air. *Curr. Pollut. Rep.* 3 1–16. 10.1007/s40726-016-0047-z

[B60] Pillai S. D. Ricke S. C. (2002). Bioaerosols from municipal and animal wastes: background and contemporary issues. *Can. J. Microbiol.* 48 681–696. 10.1139/w02-070 12381025

[B61] Polis G. A. Anderson W. B. Holt R. D. (1997). Toward an integration of landscape and food web ecology: the dynamics of spatially subsidized food webs. *Annu. Rev. Ecol. Syst.* 01 289–316. 10.1146/annurev.ecolsys.28.1.289

[B62] Pósfai M. Li J. Anderson J. R. Buseck P. R. (2003). Aerosol bacteria over the Southern Ocean during ACE-1. *Atmos. Res.* 66 231–240. 10.1016/S0169-8095(03)00039-5

[B63] Prospero J. M. Blades E. Mathison G. Naidu R. (2005). Interhemispheric transport of viable fungi and bacteria from Africa to the Caribbean with soil dust. *Aerobiologia* 21 1–19. 10.1007/s10453-004-5872-7

[B64] Quince C. Curtis T. P. Sloan W. T. (2008). The rational exploration of microbial diversity. *ISME J.* 2:997. 10.1038/ismej.2008.69 18650928

[B65] Quince C. Lanzen A. Curtis T. P. Davenport R. J. Hall N. Head I. M. (2009). Accurate determination of microbial diversity from 454 pyrosequencing data. *Nat Methods* 6 639–641. 10.1038/nmeth.1361 19668203

[B66] R Core Team (2018). *R: A Language and Environment for Statistical Computing*. Vienna: R Foundation for Statistical Computing.

[B67] Ravva S. V. Hernlem B. J. Sarreal C. Z. Mandrell R. E. (2012). Bacterial communities in urban aerosols collected with wetted-wall cyclonic samplers and seasonal fluctuations of live and culturable airborne bacteria. *J. Environ. Monit.* 14 473–481. 10.1039/C1EM10753D 22193549

[B68] Šantl-Temkiv T. Gosewinkel U. Starnawski P. Lever M. Finster K. (2018). Aeolian dispersal of bacteria in southwest Greenland: their sources, abundance, diversity and physiological states. *FEMS Microbiol. Ecol.* 94:fiy031. 10.1093/femsec/fiy031 29481623

[B69] Säwström C. Hyndes Glenn A. Eyre Bradley D. Huggett Megan J. Fraser Matthew W. Lavery Paul S. (2016). Coastal connectivity and spatial subsidy from a microbial perspective. *Ecol. Evol.* 6 6662–6671. 10.1002/ece3.2408 27777738 PMC5058536

[B70] Schloss P. Girard R. Martin T. Edwards J. Thrash J. (2016). Status of the archaeal and bacterial census: an update. *MBio* 06 e00201–e00216. 10.1128/mBio.00201-16 27190214 PMC4895100

[B71] Schloss P. D. Gevers D. Westcott S. L. (2011). Reducing the effects of PCR amplification and sequencing artifacts on 16S rRNA-based studies. *PLoS One* 6:e27310. 10.1371/journal.pone.0027310 22194782 PMC3237409

[B72] Shao Y. Chung B. S. Lee S. S. Park W. Jeon C. O. (2009). *Zoogloea caeni* sp. nov., a floc-forming bacterium isolated from activated sludge. *Int. J. Syst. Evol. Microbiol.* 59 526–530. 10.1099/ijs.0.65670-0 19244434

[B73] Tsirogiannis C. Sandel B. (2015). PhyloMeasures: a package for computing phylogenetic biodiversity measures and their statistical moments. *Ecography* 01 709–714.

[B74] Van der Gucht K. Cottenie K. Muylaert K. Vloemans N. Cousin S. Declerck S. (2007). The power of species sorting: local factors drive bacterial community composition over a wide range of spatial scales. *Proc. Natl. Acad. Sci. U.S.A.* 18 20404–20409. 10.1073/pnas.0707200104 18077371 PMC2154443

[B75] VandeWalle J. L. Goetz G. W. Huse S. M. Morrison H. G. Sogin M. L. Hoffmann R. G. (2012). *Acinetobacter, Aeromonas* and *Trichococcus* populations dominate the microbial community within urban sewer infrastructure. *Environ. Microbiol.* 14 2538–2552. 10.1111/j.1462-2920.2012.02757.x 22524675 PMC3427404

[B76] Wéry N. Monteil C. Pourcher A.-M. Godon J.-J. (2010). Human-specific fecal bacteria in wastewater treatment plant effluents. *Water Res.* 44 1873–1883. 10.1016/j.watres.2009.11.027 19945729

[B77] Wickham H. (2009). *ggplot2: Elegant Graphics for Data Analysis*. New York, NY: Springer-Verlag 10.1007/978-0-387-98141-3

[B78] Womack A. M. Bohannan B. J. M. Green J. L. (2010). Biodiversity and biogeography of the atmosphere. *Philos. Trans. R. Soc. B-Biol. Sci.* 365 3645–3653. 10.1098/rstb.2010.0283 20980313 PMC2982008

[B79] Woo A. C. Brar M. S. Chan Y. Lau M. C. Y. Leung F. C. C. Scott J. A. (2013). Temporal variation in airborne microbial populations and microbially derived allergens in a tropical urban landscape. *Atmos. Environ.* 74 391–300. 10.1016/j.atmosenv.2013.03.047

[B80] Xia X. Wang J. Ji J. Zhang J. Chen L. Zhang R. (2015). Bacterial communities in marine aerosols revealed by 454 Pyrosequencing of the 16S rRNA Gene. *J. Atmos. Sci.* 72 2997–3008. 10.1175/JAS-D-15-0008.1

[B81] Young S. Juhl A. R. O’Mullan G. D. (2013). Antibiotic-resistant bacteria in the Hudson River Estuary linked to wet weather sewage contamination. *J. Water Health* 11 297–310. 10.2166/wh.2013.131 23708577

[B82] Zhou J. Ning D. (2017). Stochastic community assembly: does it matter in microbial ecology? *Microbiol. Mol. Biol. Rev.* 81:e00002-17. 10.1128/MMBR.00002-17 29021219 PMC5706748

[B83] Zweifel U. L. Hagstrom A. Holmfeldt K. Thyrhaug R. Geels C. Frohn L. M. (2012). High bacterial 16S rRNA gene diversity above the atmospheric boundary layer. *Aerobiologia* 28 481–498. 10.1007/s10453-012-9250-6

