# Supplementary material for: Comparison of Bacterial Diversity in Air and Water of a Major Urban Center
Source: Front Microbiol. 2018 Nov 29;9:2868. doi: 10.3389/fmicb.2018.02868 (PMC6282627; doi:10.3389/fmicb.2018.02868)
Supplement: Supplementary file 1 [file Data_Sheet_1.PDF]

Supplemental Materials

Table S1. Sampling sites and meteorological context

Site	Aerosol Samples	Total aerosol sequences	Water Samples	Total water sequences	RH (%)	Wind Speed (m s^{-1})	Air Temp. (°C)	Water Temp. (°C)	Water Salinity (ppt)
Flushing Bay (FB)	15	25,168	15	37,981	42.8 ± 3.9	4.4 ± 0.5	17.9 ± 1.3	15.9 ± 1.1	20.9 ± 0.7
Louis Valentino Pier (LVP)	4	20,220	4	14,699	46.4 ± 7.6	10.0 ± 0.5	9.6 ± 0.5	8.9 ± 0.1	19.8 ± 0.1
Newtown Creek (NC)	11	27,496	6	23,154	47.8 ± 5.2	2.6 ± 0.5	18.1 ± 2.4	11.3 ± 0.9	17.7 ± 0.4

Table S2. Presence of sewage associated genera (with relevant references) in water and aerosol sequence libraries (X=present)

**Fecal/Sewage**	**Reference**	**Urban Water**	**Urban Aerosols**
Acetanaerobacterium	Newton 2015		X
Anaerofilum	Newton 2015		X
Bacteroides	Wery 2010, Korajkic 2015	X	X
Bifidobacterium	Wery 2010	X	X
Blautia	Cai 2014, McLellan 2014	X	X
Butyrivibrio	Newton 2015		X
Catenibacterium	Newton 2015	X	X
Clostridium_IV	Newton 2015		X
Clostridium_III	Newton 2015		X
Clostridium_sensu_stricto	Newton 2015		X
Clostridium_XI	Newton 2015		X
Dorea	Newton 2015		X
Dysgonomonas	Newton 2015		X
Enterococcus	Newton 2015		X
Faecalibacterium	Newton 2015	X	X
Lachnospira	Barberan 2015	X	X
Parabacteroides	Barberan 2015	X	X
Phascolarctobacterium	Barberan 2015	X	X
Roseburia	Newton 2015, Barberan 2015	X	X
Ruminococcus	Newton 2015, Barberan 2015		X
Tepidimicrobium	Newton 2015		X
Zoogloea	Shao 2009	X	X
Romboutsia	Gerritsen 2014	X	X
**Sewage Infrastructure**			
Arcobacter	Brodie 2006, Newton 2015	X	X
Aeromonas	VandeWalle 2012	X	X
Acinetobacter	VandeWalle 2012, Newton 2015	X	X
Trichococcus	VandeWalle 2012, Newton 2015	X	X

Table S3. Environmental sources from dominant sewage OTU top-hits (aerosol and water libraries combined) from GenBank BLAST (NCBI)

Sequence ID	OTU#	# of sequences	sources
>IJZ5VFS02JEJOR	Otu00017	3144	batch reactor, sludge digester, fecal contaminated water
>IJZ5VFS02JGR2J	Otu00038	1426	activated sludge wastewater, seawater, oilfield
>JJHGKRG01COP6C	Otu00040	1350	activated sludge, fish kidney, human diseased tissue
>I0J3TCE01B94YO	Otu00048	1115	activated sludge, oil reservoir, coastal waters, industrial coastal sediments, Yellow Sea, biocorroded metal
>JJHGKRG01AK3RQ	Otu00053	980	wastewater-influenced river, WWTP effluent, bird feces, activated sludge, biocorrosion of wall paintings
>IJZ5VFS02GET6X	Otu00055	959	waste water treatment plant, cow uterus, plant leaves, fecal-contaminated river water
>JJHGKRG01CT4NN	Otu00058	905	fish intestine, seawater, Yellow sea, oysters, hydrothermal sediment
>H8Y4T4E01BXJ6Q	Otu00094	614	human intestine, mouse colon
>IJZ5VFS02FWQ9W	Otu00104	505	biomass degrading microbial community (Yellowstone), oil reservoir, seawater
>IJZ5VFS02JDFGS	Otu00124	421	seawater
>IJZ5VFS02GUQEW	Otu00135	393	sludge from anaerobic digester, extraction kit contaminant, WWTP sludge, corn straw anaerobic reactor, wastewater, cow rumen
>JJHGKRG01CA8JA	Otu00140	379	rhizosphere soil, basalt, hydrothermal precipitates, lake water
>JJHGKRG01CCC0S	Otu00160	318	effluent-contaminated river water, activated sludge, radioactive waste, anaerobic reactor, metalworking fluids
>IJZ5VFS02IHII0	Otu00189	271	aerobic sludge digester, dairy products, anaerobic sediment
>IJZ5VFS02HEL77	Otu00196	255	activated sludge, sewage-contaminated urban canal, rural domestic wastewater, urban streamwater, seawater
>IJZ5VFS02HIVM3	Otu00210	244	sheep rumen, cattle rumen, human feces, calf rectum
>IJZ5VFS02JHD9S	Otu00218	237	raw milk, Baltic ice
>I0J3TCE01C9FKA	Otu00220	231	oil-contaminated soil, pig litter, rumen fluid and lake sediments, water pipe biofilm, human skin (diseased)
>IJZ5VFS02HSYS4	Otu00227	225	human feces, human skin (diseased)
>IJZ5VFS02HH2T2	Otu00250	208	activated sludge marine sediment, human skin, hot springs,
>IJZ5VFS02F72X8	Otu00254	206	human feces, human skin (diseased)
>IJZ5VFS02FNV75	Otu00256	204	wastewater biofilm, rumen, cheese shelves, oil reservoir, tropical fungi, human sinus
>IJZ5VFS02H1X84	Otu00295	169	youth intestine, mouse feces, stromatolite, human feces, human gut
>IJZ5VFS02JEZUI	Otu00322	152	domestic grey water, river, human skin, mouse skin
>IJZ5VFS02JK4HH	Otu00349	142	human feces, snow leopard feces
>IJZ5VFS02HJK0P	Otu00355	141	human adult, human feces
>IJZ5VFS02F90NY	Otu00378	128	coastal waters, marine sediment, oil-contaminated seawater
>I0J3TCE01EIWEI	Otu00391	122	salmon farm sediment, oysters, seawater, anoxic basin, Yellow Sea
>JJHGKRG01BI82P	Otu00394	121	sewage sludge, anaerobic sludge reactor
>IJZ5VFS02F6JE9	Otu00422	112	industrial effluent, human skin, eutrophic bay, leaf litter
>IJZ5VFS02HVGPM	Otu00448	104	domestic grey water, river water, human skin, mouse skin
>IJZ5VFS02FOIEO	Otu00449	104	wastewater, human skin, animal liver, rain water, raw milk
>IJZ5VFS02IYOPW	Otu00470	96	human feces
>JJHGKRG01DQ92X	Otu00471	96	hospital sewage, cheese shelving, rainwater, Sichuan kimchi, coastal sand
>IJZ5VFS02F0JB0	Otu00498	90	sewage contaminated urban canal, bioelectrochemical system, oil-field, oil reservoir, coalbed, industrial effluent, wastewater

>IJZ5VFS02JMIAO	Otu00510	87	activated sludge, sponge microbiome, concrete sewer biofilm, wastewater, fecal contaminated riverwater, anaerobic swine lagoon
>IJZ5VFS02FY4Z0	Otu00554	79	human gut, human adult, youth feces
>IJZ5VFS02IKB17	Otu00564	78	marine sediment, oysters, deep-sea vent biofilm, oil-contaminated water, microbial fuel cell with marine bacteria
>IJZ5VFS02I4POY	Otu00584	74	wastewater, anaerobic benzene H-cell, dairy pateurizer, coal sediment, oilfield, anaerobic sludge digester
>IJZ5VFS02IQA7U	Otu00594	72	human feces, human skin (diseased)
>IJZ5VFS02GBA8W	Otu00596	72	activated sludge, drinking water biofilm, peat layer, sediment, lakewater, contaminated river sediment
>IJZ5VFS02F3IQI	Otu00610	69	activated sludge, fish, water, fish gut flora, bird feces, roach gut, spoiled meat, groundwater
>IJZ5VFS02JJ3RE	Otu00680	59	MBR batch reactor, anaerobic sludge digestor, fecal-contaminated river water, wastewater sludge, rural domestic wastewater
>IJZ5VFS02HIQVH	Otu00682	59	human feces, human skin (diseased)
>IJZ5VFS02GOK49	Otu00709	56	activated sludge, Baltic Sea sediment, oysters
>IJZ5VFS02FTKPV	Otu00712	56	human feces
>IJZ5VFS02J4GZL	Otu00744	52	youth intestine, cattle rumen, human gut, mouse colon, human skin
>IJZ5VFS02G22DS	Otu00756	52	anaerobic digester, animal rumen and lake sediment
>IJZ5VFS02JHIXH	Otu00775	50	wastewater treatment sludge, contaminated aquifer sediment, sewage contaminated beach, biogas fermenter
>JJHGKRG01BKD34	Otu00785	49	ocean water, Black Sea suboxic zone, coral microbiome, oil field, seaweed, oysters

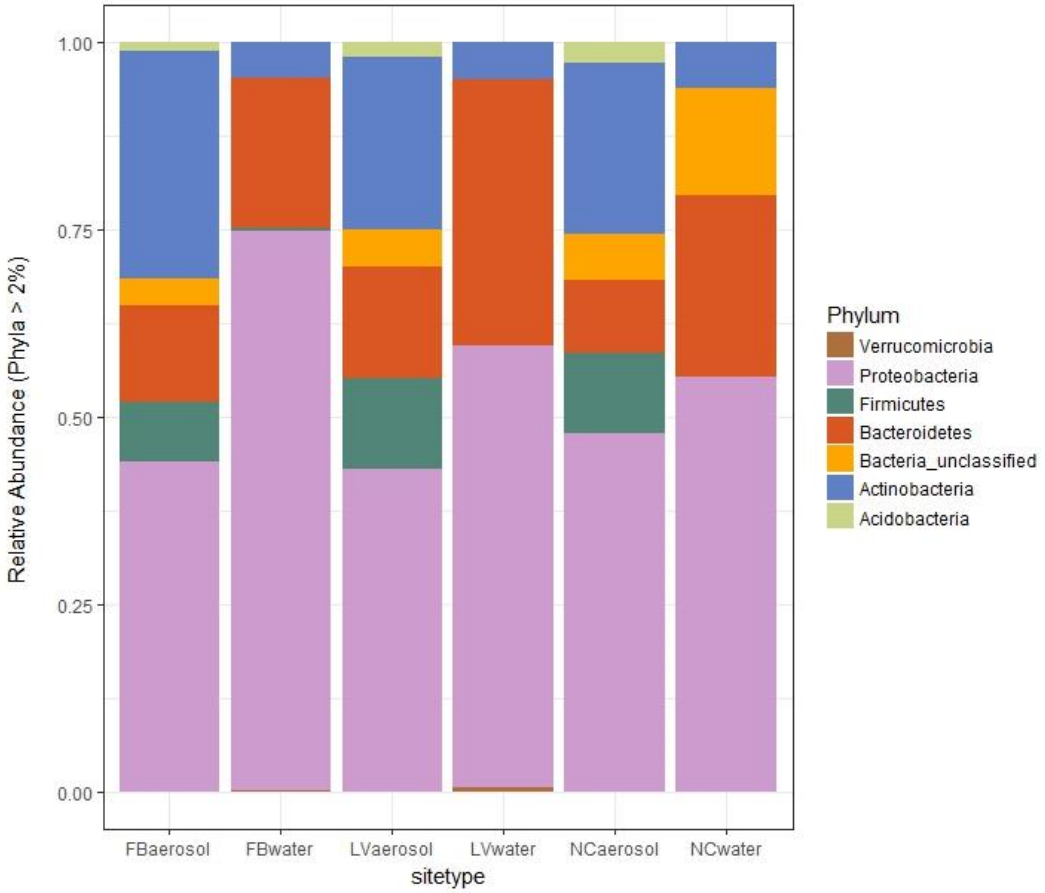

Figure S1. Phylum-level relative abundance of bacterial sequences in air and water samples.

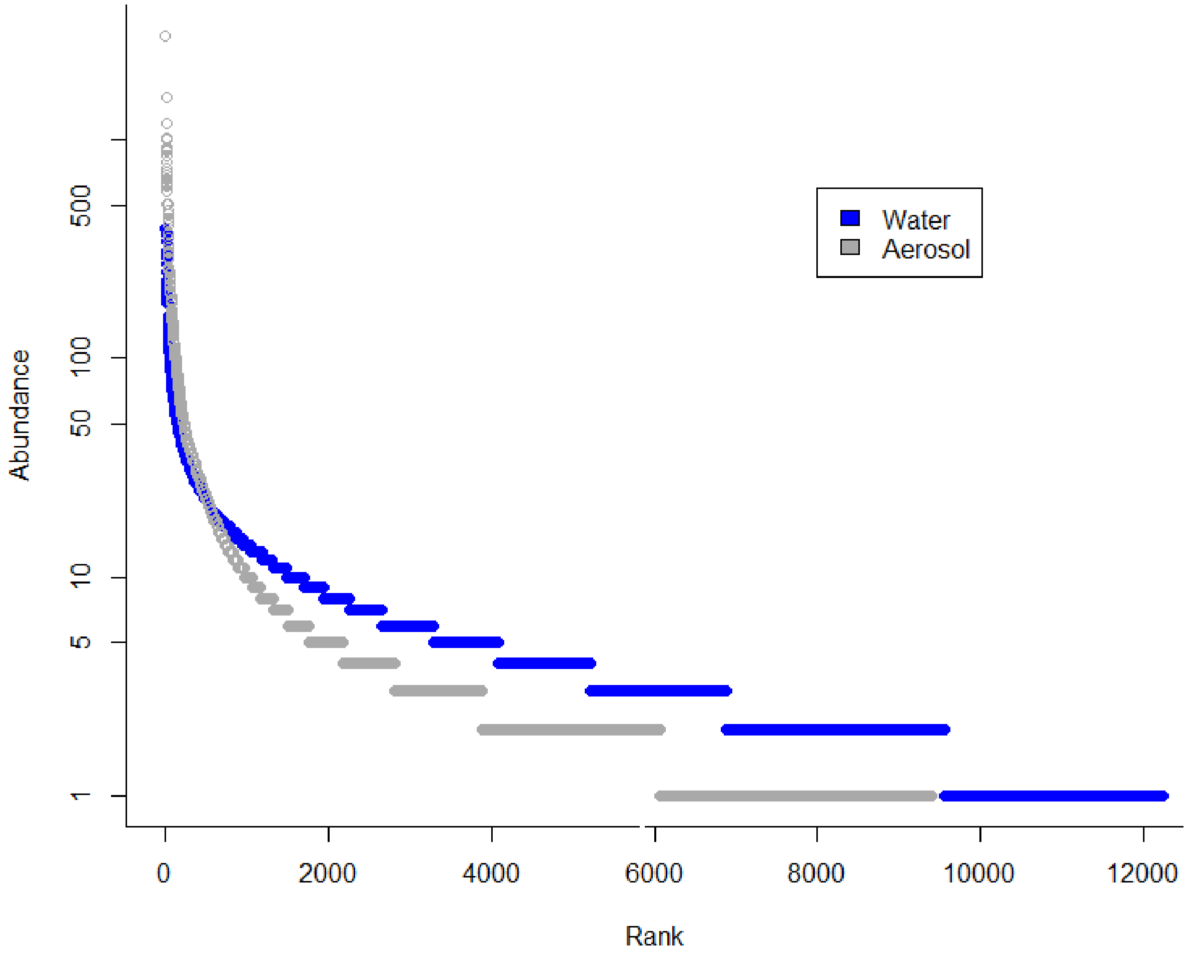

Figure S2. Rank abundance curves for aerosol and water sequences (based on OTUs at 97% identity).

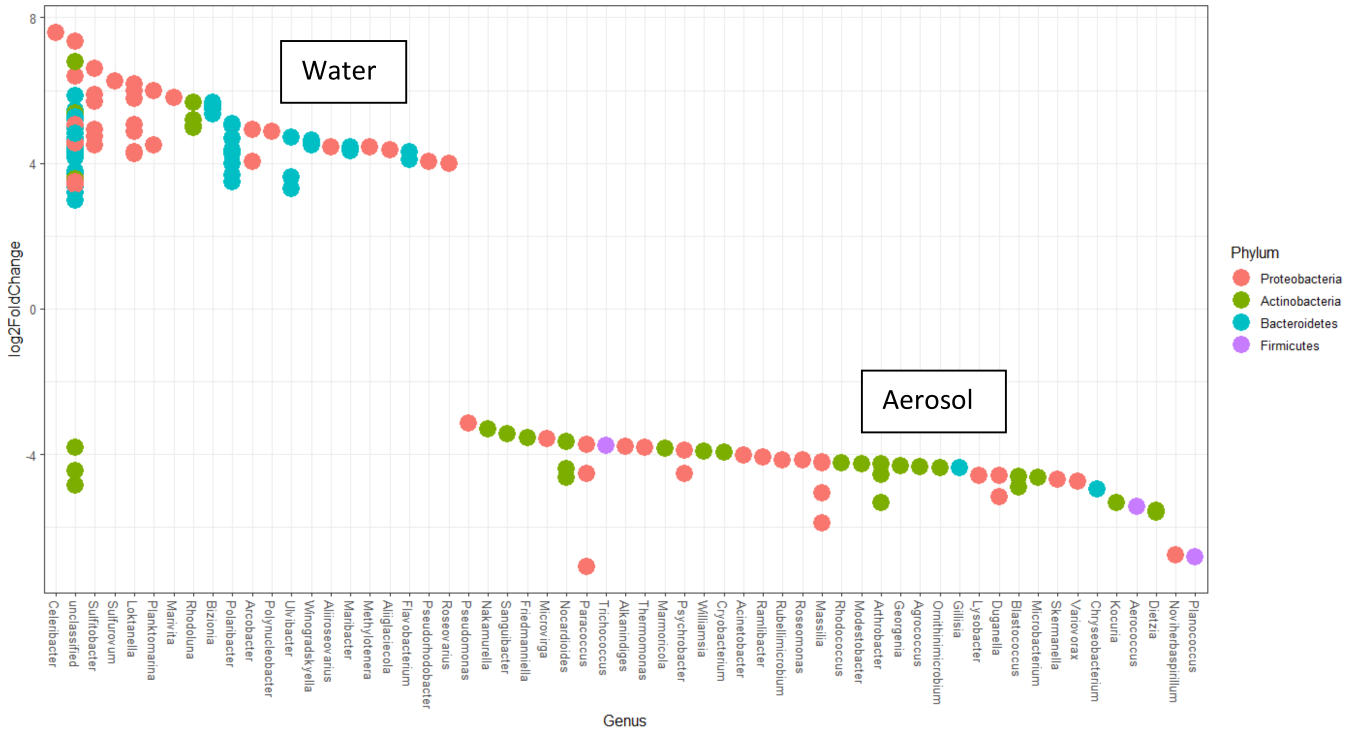

Figure S3. Differential abundance analysis (using DESeq2) identifying taxa that were considered to be “over-represented” in water vs. aerosol libraries. These can also be considered as what are unique to air and water libraries city-wide

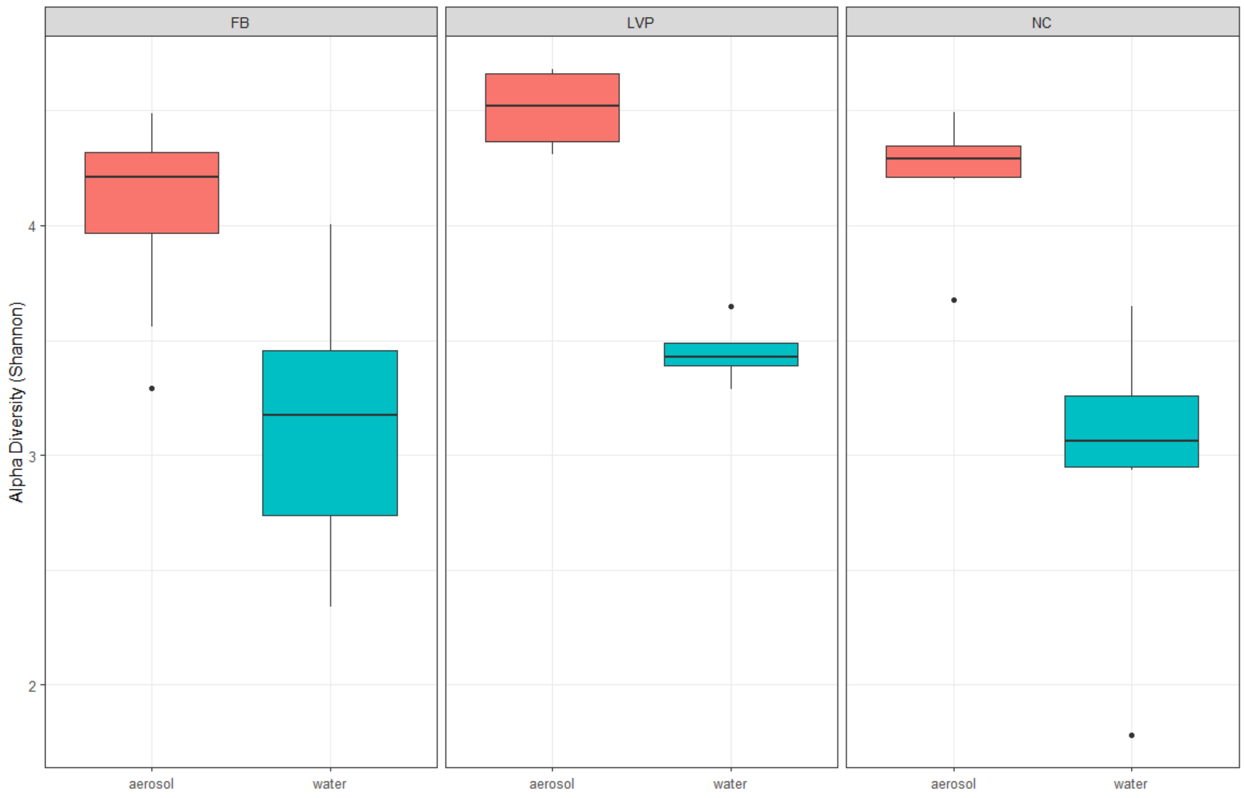

Figure S4. Alpha diversity analysis using rarefaction on sequence libraries.

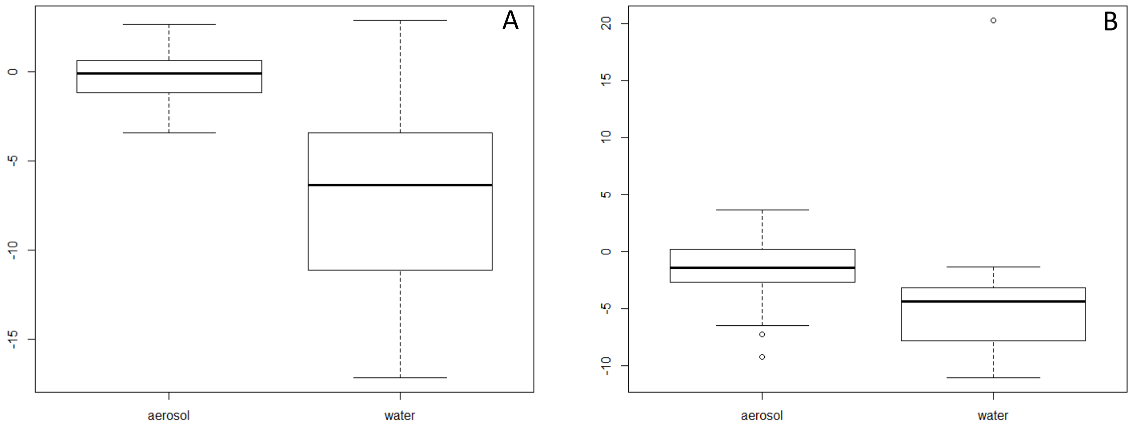

Figure S5. Non-rarefied results for (A) SES MPD analysis (mean pairwise distance) and (B) SES MNTD analysis (Mean Nearest Taxon Distance). For both indices, aerosol and water sample values were significantly different ($p < 0.01$).

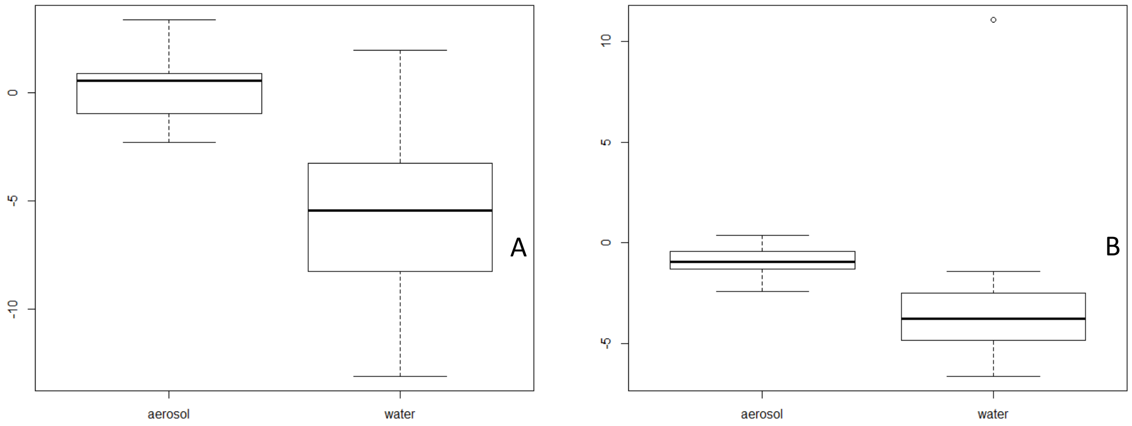

Figure S6. Rarefied results for (A) SES MPD analysis (mean pairwise distance) and (B) SES MNTD analysis (Mean Nearest Taxon Distance). For both indices, aerosol and water sample values were significantly different ($p < 0.01$).
